# Biodiversity of polycyclic aromatic hydrocarbon-degrading bacteria from deep sea sediments of the Middle Atlantic Ridge

**DOI:** 10.1111/j.1462-2920.2008.01637.x

**Published:** 2008-08

**Authors:** Zhisong Cui, Qiliang Lai, Chunming Dong, Zongze Shao

**Affiliations:** Key Laboratory of Marine Biogenetic Resources, The Third Institute of Oceanography, State Oceanic AdministrationXiamen 361005, Fujian, China

## Abstract

The bacteria involved in the biodegradation of polycyclic aromatic hydrocarbons (PAHs) in deep sea subsurface environments are largely unknown. In order to reveal their biodiversity, sediments from 2.2 m under the bottom surface at a water depth of 3542 m were sampled on the Middle Atlantic Ridge with a gravity column sampler. The sediments were promptly enriched with either crude oil or a mixture of PAHs (naphthalene, phenanthrene and pyrene) as the sole carbon source, and further enriched with the PAH mixture mentioned above in the lab. The resulting consortia were named C2CO and C2PPN respectively. Their bacterial composition was analysed with plate cultivation, PCR-DGGE and 16S rDNA library analysis. On plates, isolates belonging to *Pseudoalteromonas*, *Halomonas*, *Marinobacter*, *Thalassospira* and *Tistrella* dominated the culturable populations. With PCR-DGGE, five major bands closely related to *Cycloclasticus*, *Alteromonas*, *Thalassospira*, *Alcanivorax* and *Rhodospirillaceae* were detected in consortium C2CO, while only one major band of *Cycloclasticus* was detected in consortium C2PPN. In addition, the dynamics of community structure in response to aromatic substrate alterations were examined. As a result, three ribotypes of *Cycloclasticus* were detected by 16S rDNA library analysis, one which played a key role in phenanthrene degradation; two *Alteromonas* bacteria dominated the naphthalene reselected consortium. Although bacteria of the two genera grew as the main members of the communities, none of them were isolated, probably owing to their poor cultivability. These results confirm that bacteria of *Cycloclasticus* are important obligate PAH degraders in marine environments, and coexist with other degrading bacteria that inhabit the deep subsurface sediment of the Atlantic. This supports the view that PAH accumulation and bioattenuation occur in remote areas consistently and continuously.

## Introduction

Ocean water covers more than 70% of the earth's surface. Each year, large amounts of pollutants are introduced into the sea by land runoff and direct discharge. Oceans are undoubtedly the largest reservoirs on earth where various pollutants are deposited. Pollutant accumulation in oceans has roused great concern mainly with respect to near shore environments (http://oceanlink.island.net/ask/pollution.html). However, about 60% of the area of ocean water is over 2000 m deep (Bull *et al*., 2000). Despite their remoteness, deep sea habitats are contaminated with man-made organochlorinated compounds (Woodwell *et al*., 1971). Likely, polycyclic aromatic hydrocarbons (PAHs) also occur in remote deep-sea areas (Karinen, 1980; Lipiatou *et al*., 1997), and are accumulated by deep-sea organisms (Steimle *et al*., 1990; Escartin and Porte, 1999; Porte *et al*., 2000). Compared with our knowledge on coastal areas, the fate of pollutants in deep sea environments is less known (Deming, 1998).

On the other hand, the deep sea is an oligotrophic environment where organic pollutants are possibly scarce carbon sources fit for some heterotrophic bacteria. However, we do not know about these organisms *in situ*, considering the large and variable environment of the deep sea. Biodegradation of hydrocarbons in deep sea environments was first reported by Schwarz and colleagues (1974a,b). *Aeromonas*, *Pseudomonas* and *Vibrio* spp. had been found to be hydrocarbon degraders in Atlantic Ocean sediment samples collected at a depth of 4940 m on the continental shelf. However, the bacteria in oceanic environments involved in the degradation of organic pollutants are not known.

In previous studies, a variety of bacteria were found in a consortium from deep sea sediments of the west Pacific (2682 m). The consortium was found to degrade all tested PAH compounds with the exception of chrysene and benzo[α]pyrene, including pyrene, acenaphthene, fluorene, anthracene and fluoranthene. During the degradation of these PAHs, *Cycloclasticus* sp. P1 played an important role, and distinguished itself from other members of this genus by efficient pyrene degradation (Wang *et al*., 2008).

In this study, deep sea sediments were sampled from the middle ridge of the Atlantic, at a 3542 m water depth and over 2 m under the sea floor. Such a depth of sediment needs hundreds of thousands of years to deposit, and it should be far from pollution of modern industrialization spatially and temporally. We attempted to detect bacteria related to PAH degradation, and determine if these species were different from those from coastal. Our results will help to illustrate the role of bacteria in eliminating such persistent pollutants in extreme environments.

## Results

### PAHs in sediment

After freeze-drying, Soxhlet extraction, GC-MS quantification showed that in MAR-C2 sediment, the total concentration of the detected PAHs was 260 ng g^−1^ dw (dry weight). Seven kinds of PAHs were detected with concentrations as the following: naphthalene 38 ng g^−1^, acenaphthene 89 ng g^−1^, fluorene 17 ng g^−1^, phenanthrene 68 ng g^−1^, anthracene 12 ng g^−1^, fluoranthene 17 ng g^−1^, pyrene 19 ng g^−1^. Chrysene and Benzo(a)pyrene were not detected.

### Isolation and identification of PAH-degrading bacteria from MAR sediment

The deep sea sediment was sampled with a gravity column sampler at 0°8.42′N, 24°23.63′W on the ‘Dayang Yihao’ research vessel. The sampling site is near the cross-point of the middle Atlantic ridge with the equator, at a water depth of 3542 m, and a 2.3 m high sediment column. The sediment used was the layer at about 2.2 m depth, which contained fine grey granules. The sediment sample was enriched, respectively, with crude oil and a PAH mixture composed of naphthalene, phenanthrene and pyrene as the sole sources of carbon and energy. Obvious bacterial growth occurred in both treatments after about 2 months of incubation on board. They were then transferred twice into fresh medium with the PAH mixture as the sole carbon source in the lab, and two PAH-degrading consortia were finally obtained and named C2CO and C2PPN.

Bacteria in the two consortia were isolated with M8 plate medium and further identified by sequencing of partial 16S rDNA (about 850 bp). From the consortium C2CO, 14 isolates were subjected to sequence analysis, and finally 12 strains of different 16S rDNA sequences were obtained belonging to six genera of the *Alpha*- and *Gammaproteobacteria* (Table 1). From the community C2PPN, 13 isolates of varied colony morphology were picked, and eight were found to have different sequences; they belonged to five genera of the *Alpha-* and *Gammaproteobacteria* (Table 1). The isolates sharing identical sequence from the two consortia were paired as 1B = 1H, 1E = 1Q, 2C = 2G, 2E = 2H, 2 J = 2 V, 2F = 2T = 2 U.

**Table 1:** Bacterial isolates from two PAHs-degrading consortia enriched with MAR sediment.^a^

Strains (GenBank Accession No.)	Closest type strains in GenBank database (Accession No.)	Length of fragment for alignment analysis (bp)	Similarity (%)
1R(DQ768646)	*Alcanivorax borkumensis* SK2 (ABY12579)	860	98.26
1S (DQ768647)	*Alcanivorax dieselolei* NO1A (AY683531)	867	99.77
1G (DQ768628)	*Alcanivorax venusti* ISO4 (AF328762)	855	98.36
1P (DQ768644)	*Alcanivorax venusti* ISO4 (AF328762)	868	98.50
1T (DQ768621)	*Alcanivorax venusti* ISO4 (AF328762)	868	100
2J (DQ768657)	*Alcanivorax venusti* ISO4 (AF328762)	886	100
2S (DQ768660)	*Alcanivorax venusti* ISO4 (AF328762)	884	99.89
2V (DQ768650)	*Alcanivorax venusti* ISO4 (AF328762)	886	100
1B (DQ768623)	*Halomonas meridiana* DSM 5425 (HME306891)	867	100
1H (DQ768633)	*Halomonas meridiana* DSM 5425 (HME306891)	867	100
2F (DQ768653)	*Kaistia adipata* Chj404 (AY039817)	862	88.63
2T (DQ768661)	*Kaistia adipata* Chj404 (AY039817)	862	88.63
2U (DQ768662)	*Kaistia adipata* Chj404 (AY039817)	862	88.63
2P (DQ768658)	*Marinobacter alkaliphilus* (AB125942)	885	100
1C (DQ768624)	*Marinobacter bryozoanae* KMM 3840 (AJ609271)	867	99.08
1K (DQ768641)	*Marinobacter koreensis* KGB22 (DQ097526)	864	98.15
2E (DQ768652)	*Marinobacter vinifirmus* FB1 (DQ235263)	885	99.66
2H (DQ768655)	*Marinobacter vinifirmus* FB1 (DQ235263)	884	99.66
1A (DQ768622)	*Pseudoalteromonas ganghwensis* FR1302 (DQ011614)	865	99.42
1N (DQ768643)	*Stappia aggregate* CHLG 11 (AY639889)	840	92.74
1D (DQ768625)	*Thalassospira lucentensis* (AF358664)	836	97.01
1E (DQ768626)	*Thalassospira lucentensis* (AF358664)	844	96.45
1Q (DQ768645)	*Thalassospira lucentensis* (AF358664)	844	96.45
2C (DQ768651)	*Thalassospira lucentensis* (AF358664)	860	96.63
2G (DQ768654)	*Thalassospira lucentensis* (AF358664)	860	96.63
2I (DQ768656)	*Thalassospira lucentensis* (AF358664)	855	96.73
2R (DQ768659)	*Tistrella mobilis* (AB071665)	838	99.28

a Isolates numbered with ‘1’ before a letter were obtained from consortium C2CO, and numbered with ‘2’ from consortium C2PPN.

**Fig. 1:**
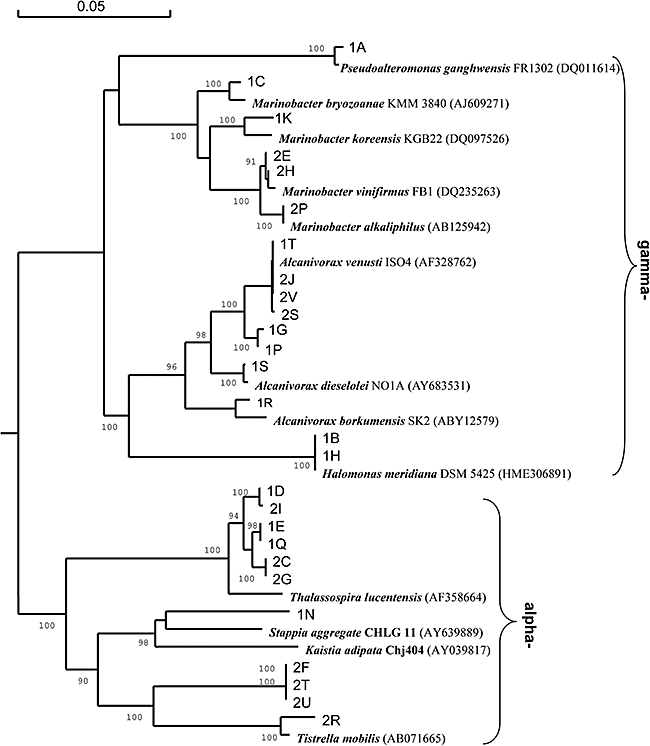
Phylogenetic analysis of the isolates from the PAH-degrading consortia C2CO and C2PPN enriched from the MAR deep sea sediment. The phylogenetic tree was constructed based on 16S rDNA gene fragments (about 860 bp). ‘1’ and ‘2’ in isolate numbers stand for consortia C2CO and C2PPN respectively. Reference strains used in the phylogenetic tree can be retrieved by the database accession number in parentheses. The numbers at the branch nodes are bootstrap values based on 1000 re-samplings for maximum likelihood. Only bootstrap values greater than 90% are shown. Scale bar equals approximately 5% nucleotide divergence.

With the partial sequences of 16S rDNA of all the isolates from the two consortia, a rooted phylogenetic tree was constructed (Fig. 1). The biggest group was *Alcanivorax*, which was composed of eight strains belonging to three species, followed by another group comprised of six isolates of *Thalassospira lucentensis* (about 96%). The two genera above occurred in both consortia, but others occurred preferentially. In more detail, bacteria close to *Alcanivorax venusti* ISO4 were isolated from both treatments, while *Alcanivorax dieselolei* NO1A (99.77%) and *Alcanivorax borkumensis* SK2 (98.26%) were isolated only from consortia C2CO that was initially enriched with crude oil. *Alcanivorax dieselolei* has been detected in both coastal areas and in Pacific deep sea sediments (Liu and Shao, 2005); *A. borkumensis* SK2 is also a ubiquitous oil degrader in marine environments (Yakimov *et al*., 1998).

Members of the genera *Pseudoalteromonas*, *Halomonas* and *Marinobacter* have been proven to be marine PAH degraders (Gauthier *et al*., 1992; Calvo *et al*., 2002; Melcher *et al*., 2002). They were also detected in the enriched cultures with the deep sea sediments used in this report. For example, in the two consortia, four isolates of different species of *Marinobacter* were obtained (Fig. 1). In addition, two novel bacteria that were distantly related with *Stappia aggregate* CHLG11 (92.74%) and *Kaistia adipata* Chj404 (88.63%) were isolated from the consortium C2PPN.

### DGGE analysis of the bacterial structure of the two consortia

**Fig. 2:**
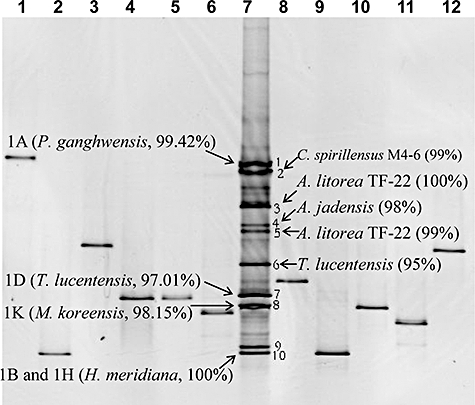
DGGE profile (20–65% denaturant) of PCR-amplified V3 region fragments of 16S rDNA gene from consortium C2CO and its isolates. PCR products for DGGE of all single isolates from this consortium were loaded. Lanes 1–6: isolate 1A, 1B, 1J, 1D, 1I and 1F; lane 7, consortium C2CO; lanes 8–12, isolate 1G, 1H, 1K, 1M and 1l. Predominant DGGE bands 1–10 correspond to bands 1-1-1-10 in the text respectively.

Consortia C2CO and C2PPN were further analysed with 16S rDNA polymerase chain reaction-denaturing gradient gel electrophoresis (PCR-DGGE) to examine the predominant bacteria responsible for PAHs degradation. From the C2CO consortium, at least 10 bands were observed in the DGGE gel profile (Fig. 2), and bands 1-1 and 1-2 were a little stronger than others. Among the bands, 1-1, 1-7, 1-8 and 1-10 matched bands derived from the isolates of 1 A (*Pseudoalteromonas ganghwensis*, 99.42%) (Fig. 2, lane 1), 1D (*T. lucentensis*, 97.01%) (lane 4), 1K (*Marinobacter koreensis*, 98.15%) (lane 10) and 1B (also 1H, *Halomonas meridiana*, 100%) (lanes 2 and 9) respectively; while the isolate 1G (*A. venusti* ISO4, 98.36%) was a relatively weak band in the community (Fig. 2, lanes 7 and 8). Those that did not match other isolates were sequenced after being excised from the gel. In this consortium, six bands including 1-2, 1-3, 1-4, 1-5 and 1-6 were further sequenced.

**Fig. 3:**
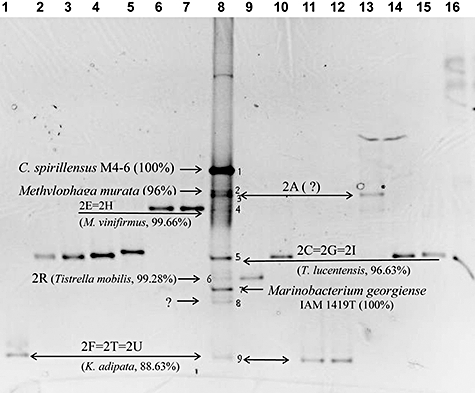
DGGE profile (20–65% denaturant) of PCR-amplified V3 region fragments of the 16S rDNA gene from consortia C2PPN and its isolates. PCR products of all single isolates from this consortium were loaded. Lanes 1–7, isolate 2F, 2B, 2I, 2J, 2P, 2E and 2H; lane 8, consortium C2PPN; lanes 9–16, isolate 2R, 2S, 2T, 2U, 2A, 2C, 2D and 2G. Predominant DGGE bands 1–9 correspond to bands 2-1-2-9 respectively. The question mark points to Band 8 that failed to be determined by both methods.

A PCR-DGGE profile of the C2PPN consortium revealed nine bands, among which only the 2-1 band represented a dominant member. However, its corresponding bacterium has not been isolated successfully. Five bands were found that matched the isolates. Among them, band 2-3 was derived from isolate 2A (16S rDNA not obtained; Fig. 3, lane 13); band 2-4 was from isolate 2E (also 2H, *Marinobacter vinifirmus*, 99.66%; Fig. 3, lanes 6 and 7); band 2-5 was from isolate 2C (also 2G and 2I, *T. lucentensis*, 96.63% and 96.73%; Fig. 3, lanes 14, 16 and 3); band 2-6 was from isolate 2R (*Tistrella mobilis*, 99.28%; Fig. 3, lane 9), and band 2-9 matched with 2F (also 2T and 2 U, *Kaistia adipata*, 88.63%; Fig. 3, lanes 1, 11 and 12). However, bands 2-2, 2-7 and 2-8 could not be retrieved from any of the isolates in the consortium.

### Bacterial identification by band sequencing

**Fig. 4:**
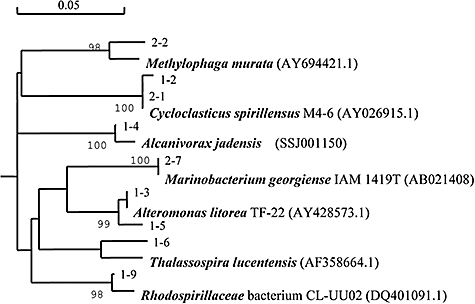
Diversity analysis based on the 195 bp long 16S rDNA gene fragments PCR amplified from the prominent bands excised from DGGE profiles of consortia C2CO and C2PPN. Numbers before ‘-’ stand for codes of the consortia. Number 1 stands for consortium C2CO, 2 stands for consortium C2PPN. Numbers after ‘-’ stand for numbering of the bands in the DGGE profiles. Type strains and their GenBank accession numbers are shown in boldface type. The numbers at the branch nodes are bootstrap values based on 1000 re-samplings for maximum likelihood. Only bootstrap values greater than 90% are shown. The scale bar equals approximately 5% nucleotide divergence.

The bands that did not match any of the isolates were subjected to DNA sequencing (Table 2, Fig. 4). Among the bands in the C2CO consortium (Fig. 2), band 1-2 was closely related to *C. spirillensus* M4-6 (99%), which was reported to be an obligate PAH degrader in coastal seawaters and sediments (Chung and King, 2001). Bands 1-3 and 1-5 were both closely related to *Alteromonas litorea* TF-22 with similarities of 100% and 98% respectively. Band 1-4 had 98.97% sequence similarity with *Alcanivorax jadensis* (SSJ001150).

**Table 2:** Sequence analyses of bands retrieved from the 16S rDNA DGGE profiles.

Bands (GenBank Accession No.)^a^	Similarity^b^	Most closely related strains in GenBank (Accession No.)
1-2 (DQ768694)	99%(193/194)	*Cycloclasticus spirillensus* M4-6 (AY026915.1)
1-3 (DQ768695)	100%(194/194)	*Alteromonas litorea* TF-22 (AY428573.1)
1-4 (DQ768697)	98%(192/194)	*Alcanivorax jadensis* (SSJ001150)
1-5 (DQ768698)	98%(191/194)	*Alteromonas litorea* TF-22 (AY428573.1)
1-6 (DQ768701)	95%(162/169)	*Thalassospira lucentensis* (AF358664.1)
1-9 (DQ768708)	98%(167/169)	*Rhodospirillaceae* bacterium CL-UU02 (DQ401091.1)
2-1 (DQ768702)	100%(194/194)	*Cycloclasticus spirillensus* M4-6 (AY026915.1)
2-2 (DQ768709)	96%(188/194)	*Methylophaga murata* (AY694421.1)
2-7 (DQ768704)	100%(194/194)	*Marinobacterium georgiense* IAM 1419T (AB021408)

a Numbers before ‘-’ stand for codes of the consortia. Number 1 stands for consortium C2CO, 2 stands for consortium C2PPN. Numbers after ‘-’ stand for numbering of the bands in the DGGE profiles.

b Identical nucleotide (bp)/length for alignment (bp).

In community C2PPN, four bands were sequenced, including bands 2-1, 2-2, 2-7 and 2-8. Band 2-1 was revealed to be *C. spirillensus* M4-6 (100%) (Fig. 3, lane 8). In addition, bands 2-2 and 2-7 were analysed to be *Methylophaga murata* (96%) and *Marinobacterium georgiense* (100%) respectively. But band 2-8 could not be sequenced. In both consortia, *C. spirillensus* was present as a strong band, but they varied in 16S rDNA sequence with 99.48% similarity (only one base pair difference in 192 bp). Moreover, the one that was predominant in the C2PPN consortium seemed to play a more important role in PAH degradation.

### Isolation of the main degraders in *Cycloclasticus*

To isolate the *Cycloclasticus* bacteria that occurred as a major band in the DGGE profile, enriched cultures were spread on M8 medium plates, and supplied with naphthalene vapor as a carbon source. As a result, nine bacteria that were not isolated before were obtained from the two consortia; but again, bacteria belonging to *Cycloclasticus* were not included. The newly isolated bacteria are listed in Table 3, among which *Sphingobium*, *Novosphingobium*, *Halomonas* and *Pseudomonas* are all potential PAH degraders.

**Table 3:** Additional bacterial isolates from two PAHs-degrading consortia enriched with MAR sediment.

Isolates (GenBank Accession No.)	Most closely related type strains in GenBank database	Length of fragment for alignment analysis (bp)	Similarity (%)
MARC2PPND (EU019949)	*Tistrella mobilis* (AB071665.1)	858	99
MARC2PPNK (EU019950)	*Sphingobium estrogenivorans* (DQ855413)	848	96
MARC2PPNL (EU019951)	Rhodospirillaceae bacterium PH30 (AF513476)	966	99
MARC2PPNM (EU019952)	*Halomonas ventosae* (AY268080)	912	98
MARC2PPNN (EU019953)	*Pseudomonas aeruginosa* (EF151192)	894	100
MARC2PPNO (EU019954)	*Novosphingobium pentaromativorans* (AF502400)	971	99
MARC2COF (EU019955)	*Crassostrea virginica* (AF246614)	841	95
MARC2COI (EU019956)	*Alcanivorax venusti* (AF328762)	913	94

### Dynamic changes of community structure in response to different PAHs

To observe the response of bacteria in the two consortia to different PAHs, the cultures of the PAH mixture-enriched consortia were transferred to fresh media with naphthalene and phenanthrene respectively. Incubation was conducted as above, and the cultures were sampled at intervals over 21 days. The dynamic changes in the main populations in the two consortia were monitored by DGGE and further analysed by 16S rDNA gene library sequencing.

#### Naphthalene selected consortium C2CO

**Fig. 5:**
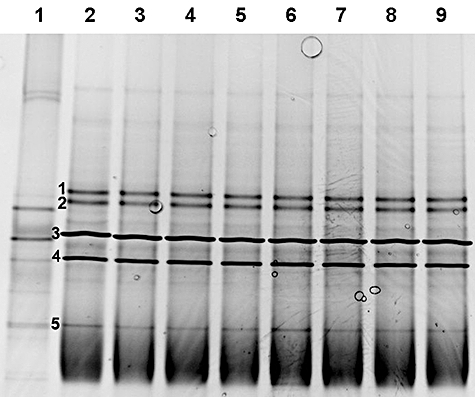
Dynamics analysis by DGGE of bacterial community C2CO transferred to medium with naphthalene as the sole source of carbon and energy. Lane 1, community C2CO after two transfers in PAH mixture used as the inoculum; lanes 2–9, community C2CO-NAH transferred to naphthalene sampled at days 1, 3, 7, 9, 11, 13, 17 and 21.

Under the selective pressure of naphthalene, consortium C2CO turned into a new consortium named C2CO-NAH. The DGGE profile confirmed that changes occurred, and indeed, the bacterial diversity was sharply reduced (Fig. 5). Most of the dominant bands of the original consortium C2CO (Fig. 2) disappeared. Instead, two new bands appeared as dominant members (1N-3 and 1N-4, numbered as bands 3 and 4 in Fig. 5). Sequence comparison showed that band 1N-3 in Fig. 5 was identical in sequence with band 1-3 in Fig. 2, while band 1N-4 in Fig. 5 only had a 2 bp variation with band 1-5 in Fig. 2.

From the 16S rDNA library of the new community, 48 clones were sequenced, 41 of which shared relatively low similarity with *Alteromonas marina* SW-49 (94%, 1400 bp/1475 bp), while others exhibited 99% similarity with *A. marina* SW-49 (1468 bp/1475 bp). By sequence alignment, they matched well with band 3 and band 4 in Fig. 5 respectively.

#### Phenanthrene selected consortium C2CO

**Fig. 6:**
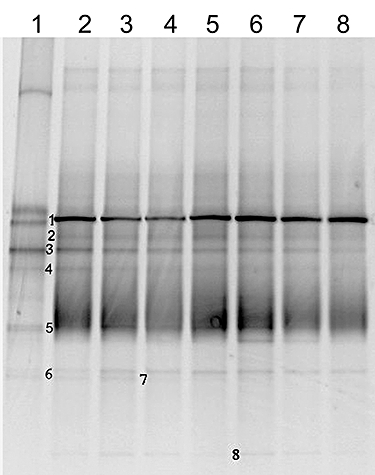
Dynamics analysis by DGGE of bacterial community C2CO transferred to medium with phenanthrene as the sole source of carbon and energy. Lane 1, community C2CO after two transfers in PAH mixture used as the inoculum; lanes 2–8, community C2CO-PHE transferred to phenanthrene sampled at days 3, 7, 9, 11, 13, 17 and 21.

Selected with phenanthrene, the bacterial diversity of the new consortium C2CO-PHE was also reduced significantly. Phenanthrene promoted only one bacterium to grow (band 1, Fig. 6). Similar to *A. marina* in naphthalene treatment, the bacterium of band 1 dominated the community C2CO-PHE during the entire process of incubation. In the 16S rDNA library of the community, 43 of 48 clones were closely related to *Cycloclasticus pugetii* PS-1 (99% similarity, 1442 bp/1449 bp), as they generated band 1 in Fig. 6. Others were too weak to be analysed.

#### Consortium C2CO reselected with PAH mixture

**Fig. 7:**
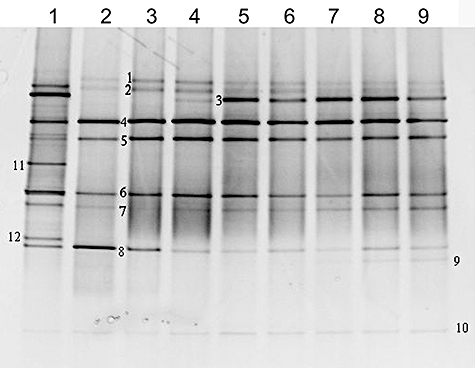
Dynamics analysis by DGGE of bacterial community C2CO transferred to PAH mixture as the sole source of carbon and energy. Lane 1, community C2CO after two transfers in PAH mixture used as the inoculum; lanes 2–9, community C2CO transferred to PAHs mixture sampled at days 1, 3, 7, 9, 11, 13, 17 and 21. DGGE bands 1–8 correspond to bands 1X-1–1X-8 in the text respectively.

Enriched with the PAH mixture for the second time, the C2CO consortium was monitored for changes in its community structure over time. A dynamic variation was observed in the first 9 days (Fig. 7, lanes 2–5). This was different from that of single PAH treatments, which easily became steady and simplified. Nine days afterwards, the stable community had a similar structure as the original C2CO (Fig. 7, lane 1). In more detail, the bacterium of band 1X-4 (4 in Fig. 7) and the bacterium of band 1X-8 (8 in Fig. 7) grew into two key members during the first day (lane 2). Band 1X-4 was closely related to *Alteromonas* sp. SHY1-1 (99%); band 1X-8 represented a novel bacterium distantly related to *Sphingosinicella microcystinivorans* MDB3 (1301 bp/1468 bp, 88%, result of following library analysis). At day 3, band 1X-5 became the main band and remained strong thereafter. Alternatively, band 1X-8 was reduced from day 3 and remained as a minor band afterwards. Around day 7, band 1X-6 (*T. lucentensis*, 99%) turned into a major band, and only subdominant to bands 1X-4 and 1X-5. At day 9, band 1X-3 turned into a main band abruptly and remained stable thereafter; it was confirmed as *C. pugetii* PS-1 (99%). In addition, there was a weak band 1X-7 present from the first day to day 21, which was identified as *Salipiger bermudensis* HTCC2601 (882 bp/897 bp, 98%).

From the 16S rDNA library of this community at day 11 (Fig. 7, lane 6), 58% clones were *Alteromonas marina* SW-49 with a similarity of only 98% (1452 bp/1475 bp). It occurred in the DGGE profile as band 1X-4. Noticeably, this was a different strain from the one in the naphthalene selection (band 1N-3) although they shared the same sequence in the DGGE-detected region. *Alteromonas marina* SW-49 (99%, 1468 bp/1475 bp) occurred as band 1X-5, which was the same bacterium as in the naphthalene selection (band 1N-4).

Interestingly, two strains of *C. pugetii* PS-1 (99%) were found from the library, with only one base pair variation (1442 bp and 1443 bp/1449 bp). Both occurred as band 1X-3 in the DGGE profile, and the first (1442 bp/1449 bp) was the same as the one in the phenanthrene treatment (Fig. 6).

#### Naphthalene selected consortium C2PPN

**Fig. 8:**
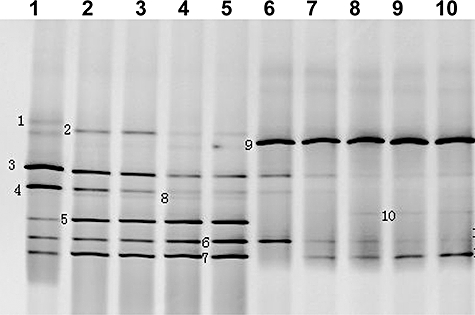
Dynamics analysis by DGGE of bacterial community C2PPN transferred to naphthalene and phenanthrene, respectively, as the sole sources of carbon and energy. Lanes 1–5, community C2PPN-NAH transferred to naphthalene sampled at days 1, 3, 5, 7 and 9. Lanes 6–10, community C2PPN-PHE transferred to phenanthene sampled at days 1, 3, 5, 7 and 9. DGGE bands 1–8 correspond to bands 2N-1–2N-8 in the text respectively. DGGE bands 9 and 10 correspond to bands 2P-9 and 2P-10.

In the case of consortium C2PPN, similar results were obtained. After transfer to medium with naphthalene as the sole carbon source, the structure of community C2PPN changed dynamically (Fig. 8, lanes 1–5). At day 1, the bacteria represented by bands 2N-3 and 2N-4 (the same as 1N-3 and 1N-4 respectively) dominated the community, but became gradually weaker from the third day. Meanwhile, the bacteria of bands 2N-5, 2N-6 and 2N-7 grew gradually stronger. At day 9, the bacteria of band 2N-5 (*Halomonas* sp. M394, 99%), 2N-6 (*T. lucentensis*, 99%) and 2N-7 (*Tistrella mobilis*, 99%) dominated the consortium. Other minor bands such as 2N-1, 2N-2 and 2N-8 were too weak to be detected.

#### Phenanthrene selected consortium C2PPN

When transferred to medium with phenanthrene as the sole carbon source, the structure of community C2PPN was simple – only one main band numbered as 2P-9 occurred and remained dominant through the entire process (Fig. 8, lanes 6–10). From the 16S rDNA library, 94% of the clones shared 99% similarity with that of *C. pugetii* PS-1 in the 1449 bp fragment. Interestingly, three ribotypes of *C. pugetii* were found: one having a 6 bp difference with strain PS-1 occupied 75% of the clones of *C. pugetii*; one having a 7 bp difference was shared with 8% of the clones of *C. pugetii*, and the one with an 8 bp difference was shared with 17% of the clones of *C. pugetii*. They had the same sequence within the V3 variable region and therefore formed one DGGE band, 2P-9 in Fig. 8.

Additionally, with the selection of phenanthrene, the two bands 2N-6 and 2N-7 in the naphthalene treatment were also present, but they showed an alternation in population: the bacterium of band 2N-6 was lost from the third day and instead, the bacterium of band 2N-7 grew stronger gradually (Fig. 8, lanes 6–10).

## Discussion

The Mid-ocean ridge is intriguing for its hydrothermal activity and unique ecosystems around the vents. On the Mid-Atlantic Ridge (MAR), several thermal vent fields have been found, such as the Lost City hydrothermal vent field (30°N), Rainbow vent field (36°14′N), and Logatchev vent field (14°45′N). What is interesting and closely related to this study is that hydrocarbons can be formed by both abiotic and biogenic processes in vent systems (Simoneit, 1990; Holm and Charlou, 2001; Foustoukos and Seyfried, 2004; Proskurowski *et al*., 2008). Although no vent has been found near the sampling site of this report, the influence of vent activity can not be excluded considering the long geographic history and the unexploited surroundings. The sediment was estimated to be a natural deposit roughly estimated at 315 000 years, according to the sedimentary rate of 0.73 cm ka^−1^ at the Ridge crest of Northern Atlantics (20°N) (Kennish, 2000). GCMS quantification proved the existence of PAHs in deep sea sediment with a total concentration comparable to that of sediments from mudflat and mangrove at Mai Po Marshes of Hong Kong (260–373 ng g^−1^ dw) (Liang *et al*., 2007). In our deep sea sediment, seven kinds of PAHs were detected with acenaphthene and phenanthrene as the most abundant two.

In marine environments, bacteria of the genus *Cycloclasticus* are obligate PAH degraders (Dyksterhouse *et al*., 1995; Kasai *et al*., 2002; Maruyama *et al*., 2003; Head *et al*., 2006). Recently, bacteria of *Cycloclasticus* were found to be important PAH degraders in seawater collected at Thames estuary (UK) spiked with crude oil or PAHs (McKew *et al*., 2007a,b; Coulon *et al*., 2007). In addition, other PAH degraders from coastal marine environments have been also reported as bacteria belonging to *Marinobacter* (Gauthier *et al*., 1992), *Neptunomonas* (Hedlund, 1999), *Sphingomonas* (Gilewicz *et al*., 1997; Kazunga and Michael, 2000;Demaneche *et al*. 2004), *Flavobacterium* (Okpokwasili *et al*., 1984), *Vibrio* (West *et al*., 1984), *Pseudoalteromonas*, *Marinomonas* and *Halomonas* (Melcher *et al*., 2002; Garcia *et al*., 2005).

In contrast with extensive studies of coastal environments, bacteria in deep sea environments responsible for PAH degradation are largely unknown. In this report, bacteria of *Cycloclasticus* were found to be an important group of PAH degraders in deep Atlantic sediment 2.2 m below the sea floor at a water depth of 3542 m, and were confirmed as the main degraders of phenanthrene in an enriched culture. In a previous study, we revealed *C. spirillensus* (100%) from the deep sea sediment of the west Pacific as a pyrene-degrader. In this report, Atlantic sediment from the middle ridge also contained *Cycloclasticus.* But the consortium was not as efficient as that from the Pacific sediment for pyrene degradation, as no growth was observed after 2 weeks, and only a slight growth with pyrene was found 1 month later. Consequently, the consortium challenged with pyrene failed to be analysed for its bacterial structure.

In addition to *Cycloclasticus*, bacteria related to *A. marina* were found as PAH degraders in the ridge sediment, and they were especially involved in naphthalene degradation. *Alteromonas marina* is a novel marine bacterium isolated from sea water from the East Sea in Korea (Yoon *et al*., 2003), but to our knowledge, no bacteria in this genus have been reported as naphthalene degraders. However, the bacteria detected in this report have not been successfully isolated. In addition, bacteria related to the genera of *Tistrella*, *Thalassospira* and *Kaistia* in the ridge sample are also potential PAH-degraders.

Regarding the coldness and high pressure of the deep sea, the bacteria and their behaviour *in situ* must be different from that in the lab. The rate of biodegradation *in situ* should be slower than that in mesophilic environments. Moreover, biodegradation is possibly the only mechanism for decomposition, as the darkness of the deep sea absolutely excludes light decomposition. Pressure is a key environmental factor in the deep sea. We do not know the details of the degradation process under high hydrostatic pressure. Cell morphology, PAH metabolic activity and even PAH absorption would change to adapt to such levels of pressure. These suggestions are supported by results with piezophilic bacteria, which are capable of changing their respiratory system in response to pressure conditions (Kato *et al*., 2000), and are regulated at the gene level in the synthesis of membrane fatty acids and proteins (Bartlett, 2000). The detected bacteria are certainly piezo-tolerant, but more efforts are needed to investigate their piezo-behaviour by mimicking *in situ* conditions in the deep sea. In addition, low temperature might select a community different from the currently found. But the growth rate is very slow at temperature below 4°C. Enrichment at this condition was conducted in parallel but has not been subjected to further analysis by now.

In summary, *Cycloclasticus* and *Altermonas* were detected as key degraders of phenanthrene and naphthalene in deep sea sediments of the middle ridge of the Atlantic Ocean. They and other potential degrading bacteria coexist in Atlantic subsurface sediments. These results indicate that PAH accumulation and bioattenuation occurred concomitantly in oceanic subsurface environments in the past, and will likely continue in the future. Insights into their adaptation and activities *in situ* will require further efforts.

## Experimental procedures

### Sediment sampling

The deep sea sediment was collected during the cruise of DY105 of R/V ‘Da-Yang Yi-Hao’ on 31 October 2005, with a gravity core sampler at a depth of 3542 m at station MAR-C2 (0°8.42′N, 24°23.63′W), which is near the cross-point of the middle Atlantic ridge with the equator. The sediment core was about 2.3 m high, 15 cm in diameter, with estimated temperature 0–1°C. It was very tightly trapped in the core tube after impact, and intact from the disturbance of overlaying seawater by an automatic mechanism closing the ends. Sediment in the core was dry (no fluid, or muddy), highly pressed and in form of grayish fine granules. The sediment in the central part of core at about 2.2 m depth from the top was cut with sterilized knives and put into a sterilized bottle and used immediately for enrichment on board after sampling. The *in situ* oxidation-reduction potential was not determined; oxygen is probably limited at this depth, but not absolutely anoxic.

### PAH compounds

Pyrene was purchased from Sigma Chemical (St Louis, Mo., 98% pure); phenanthrene was purchased from Fluka (> 97% pure); and naphthalene was purchased from Sinopharm Chemical Reagent (Shanghai, China, 99.8% pure).

### Medium

Mineral medium, which contained (per litre) 1 g of NH_4_NO_3_, 0.8 g of KH_2_PO_4_, 0.2 g of K_2_HPO_4_ and 2.8 × 10^−3^ g of FeSO_4_, dissolved in deep sea water sampled from the Ridge, pH 7.6, autoclaved for 30 min at 121°C. FeSO_4_ was sterilized with a 0.22 μm pore filter and added separately. This was used for enrichment of PAH-degrading microorganisms.

M8 medium was designed in this study, which contained (per litre) 2.0 g of CH_3_COONa, 2.0 g of NH_4_NO_3_, 0.5 g of tryptone, 0.5 g of yeast extract, 0.5 g of potato extract, 0.2 g of sucrose, 0.2 g of glucose, 0.05 g of sodium citrate, 0.05 g of malic acid, 0.05 g of potassium sodium tartrate, dissolved in 1 L of sea water (pH 7.6). This was used for cultivation of microorganisms from the enriched culture.

### Extract of PAHs from the sediment sample

The sediment was frozen at 20°C on board and then transferred to −80°C in lab. Before Soxhlet extraction, the sediment was freeze-dried with trap temperature −98°C. Then, 10 g freeze-dried sediment was placed in Soxhlet extractor and extracted with 300 ml solvent of an acetone and dichloromethane mixture (1:1 v/v), at 60°C for 24 h. The solvent extract was dehydrated by anhydrous sodium sulfate and then concentrated to 1–2 ml by vacuum rotary evaporator.

Sulfur clean-up was carried out according to US EPA method 3660B; florisil Clean-up referring to method 3620B. Both extraction and the following quantification were repeated in triplicates.

### Quantification of PAHs with GC/MS

The method was according to the US EPA method 8270D. A Shimadzu QP2010 GC-MS system equipped with a GC column (DB-5ms 30 m long, 0.25 mm internal diameter, 0.25 mm coating) was used for identification and quantification of PAH compounds.

High purity helium was used as the carrier gas with a total flow of 9 ml min^−1^ and an on-column flow of 1.30 ml min^−1^, the split ratio was 3.0 and the solvent cut time was 4.0 min. The initial oven temperature was 80°C and programmed from 80°C to 280°C at a rate of 10°C min^−1^, then hold 5 min. Electron ionization at 70 eV was used. The injector and interface temperatures were maintained at 280°C and 200°C respectively. The temperature of ion source was 250°C.

Selected ion monitoring mode was used for quantification of PAH, and Full-scan mode (SCAN) mode was used for identification of PAH by using the GC-MS Postrun Analysis software (Version 2.10, SHIMADZU).

Quantification of each kind of PAH was carried out by the external standard method by using a series of dilution of nine PAHs standard mixture. A reagent blank was run to check the interference caused by condensed solvent.

To access the recovering efficiency, a paralleled experiment was carried out by adding certain amount PAHs mixture into the freeze-dried sediment, the recovery ratio of each PAH was calculated after Soxhlet extraction and GC-MS quantification.

### Enrichment of PAHs-degrading consortia and isolation of PAHs-degraders

About 5 g sediments were added to 400 ml of mineral medium, and supplied with 0.5 ml of sterilized crude oil or PAH mixture solution. A PAH mixture of naphthalene, phenanthrene and pyrene was dissolved in chloroform and filtered through a 0.22 μm pore film and added at final concentrations of 0.5, 0.2 and 0.1 g l^−1^ respectively. The solvent was allowed to evaporate on a rotary shaker before adding to the sample or inoculation. Enrichment was conducted on board at 25°C and kept from light for about 2 months before it was shipped to the lab. The cultures of the two treatments were transferred with 1% inoculum to 100 ml fresh medium with PAH mixture as the carbon source, and repeated two times every 2 weeks. Finally, two PAHs-degrading consortia were obtained. About 10^−4^, 10^−5^ and 10^−6^ dilutions of the two cultures were spread on M8 agar plates and incubated at 25°C. Colonies that were different in morphology were streaked onto fresh M8 plates to obtain pure cultures.

### PCR amplification of the 16S rDNA genes and sequencing

Genomic DNA was prepared with a method described as Sambrook and colleagues (1989).The 16S rDNA genes were amplified from genomic DNA using the universal primer set 27f and 1502r. The thermal cycling parameters were a 5 min hot start at 95°C, followed by 32 cycles of denaturation for 1 min at 94°C, annealing at 55°C for 1 min, and extension for 1.5 min at 72°C, with a final extension of 20 min at 72°C. The PCR products were purified by an Omega Cycle-Pure PCR purification kit and sequences were determined directly using conserved bacterial 16S rDNA sequencing primers by Shanghai Invitrogen.

### Phylogenetic analysis

The 16S rDNA sequences were aligned with published sequences from the GenBank database using the NCBI BLASTN comparison software. Phylogenetic trees were constructed by the neighbour-joining method using the DNAMAN software (version 5.1, Lynnon Biosoft, Quebec, Canada). Nearly full-length 16S rDNA sequences of the most phylogenetically related strains were selected from the GenBank database as reference strains. Bootstrapping analysis was performed with 1000 re-samplings.

### DGGE analysis of the structures of the bacterial communities

Total DNA was extracted from the plateau phase bacterial consortia of the second transfer in PAHs mixtures. PCR was performed with the total DNA of the consortia or the isolates as templates. Primers DGGEf and DGGEr were used to amplify the variable V3 region of bacterial 16S rDNA genes for DGGE analysis (corresponding to positions 341–534 in the *Escherichia coli* 16S rDNA sequence). The sequence of the former primer, DGGEf, was 5′-CGCCCGCCGCGCGCGGCGGGCGGGGCGGGGGCACGGGGGGCCTACGGGAGGCAGCAG-3′, which contained a GC clamp linked to the 5′ end; the sequence of the reverse primer, DGGEr, was 5′-ATTACCGCGGCTGCTGG-3′ (Muyzer *et al*., 1993). One unit of r*Taq* (Takara) was used in a 50 μl reaction solution. The PCR procedure was as follows: an initial cycle of 5 min at 95°C, followed by 20 cycles of 45 s at 94°C, 1 min at 65°C, with a touchdown of 0.5°C per cycle and 45 s at 72°C, then followed by 16 cycles of 45 s at 94°C, 1 min at 55°C and 45 s at 72°C, with a final extension of 10 min at 72°C. Four tubes with the total DNA of the consortia as templates were conducted to minimize the bias in amplification and were concentrated to 10 μl for ease of loading and to create a better band profile.

Both the PCR products of the isolates and the consortia were loaded on the same gel for DGGE analysis with a Dcode Universal Mutation Detection system (Bio-Rad Laboratories, Hercules, CA). Electrophoresis was performed at 60°C in an 8% (w/v) polyacrylamide gel with a denaturant gradient ranging from 20% to 60% for 15 min at 30 V, then 4.5 h at 130 V in 1× Tris-acetate-EDTA buffer. After electrophoresis, the gel was incubated for 30 min in EB stain solution and the pictures were captured by an Alphalmager Imaging System with AlphaEase FC image software 4.1.0 (Alpha Innotech).

Bands of interest on the DGGE gel were excised, transferred to a clean Eppendorf tube and smashed to release the DNA into 20 μl of sterile deionized water. With 2 μl of the eluted DNA as the template, PCR was performed to generate more target DNA for cloning. The thermal cycling parameters were as follows: 5 min at 95°C, 32 cycles of 45 s at 94°C, 1 min at 55°C and 45 s at 72°C, followed by 10 min final extension at 72°C. The purity of the PCR products was further examined by DGGE electrophoresis. The PCR products, which migrated at the same position with the target DNA were purified by the EZNA Cycle-Pure Kit (OMEGA Bio-tek, USA), cloned into the pMD19-T Vector (Takara) and sequenced with an ABI model 3730 DNA sequencer (Invitrogen, Shanghai, China). The sequences were analysed as described above.

### Dynamics of bacterial communities in different PAHs as the sole source of carbon and energy

After two transfers in PAHs mixture, the cultures of C2CO and C2PPN were transferred to 500 ml deep sea water medium supplemented with 1 g l^−1^ naphthalene, 0.4 g l^−1^ phenanthrene or PAHs mixture (at the same concentration as described above) as the sole source of carbon and energy. The cultures were sampled successively to monitor community shifts. Each time, 3 ml of culture was centrifuged to collect bacterial cells for community DNA extraction. For consortia transferred from C2CO, bacterial cells were collected at days 1, 3, 7, 9, 11, 13, 17 and 21. The consortium C2CO-PHE, which was transferred to phenanthrene, was collected at day 3 when red colouring was present in the culture. For consortium C2PPN, bacterial cells were collected at days 1, 3, 5, 7 and 9. The V3 region of the bacterial 16S rDNA genes was PCR amplified for DGGE analysis as described above. Bands of interest in the DGGE profile were subjected to excision, re-amplification, cloning, sequencing and phylogenetic analysis.

To reconfirm the results of DGGE, a DNA library of the communities was constructed with nearly full-length 16S rDNA, amplified with the primer for 16SF (positions 8–27; 5′-AGAGTTTGATCCTGGCTCAG-3′) and 16SR (positions 1512−1493, *E. coli* numbering; 5′-ACGGCTACCTTGTTACGACT-3′). According to the DGGE results, only one time point was used for the library analysis. Forty-eight clones of each community were first DGGE analysed in comparison with the community, then subjected to sequencing. The percentage of each ribotype was calculated.

### Nucleotide sequence accession numbers

The partial sequences of 16S rDNA of the isolates from the PAH-degrading consortia are available from the GenBank nucleotide sequence database: DQ768621 to DQ768662, and EU019949 to EU019956. The partial 16S rDNA sequences of the unculturable strains are available under the following accession numbers: DQ768694 to DQ768709.
